# Hematologic and clinical chemistry reference intervals for six species of wild birds frequently rescued in the Republic of Korea

**DOI:** 10.3389/fvets.2024.1484082

**Published:** 2025-01-08

**Authors:** Myeongsu Kim, Zun Zun Wut Hmohn, Woochan Jang, Geonwoo Baek, Jae-Ik Han

**Affiliations:** ^1^Laboratory of Wildlife Medicine, College of Veterinary Medicine, Jeonbuk National University, Iksan, Republic of Korea; ^2^Jeonbuk Wildlife Center, Jeonbuk National University, Iksan, Republic of Korea

**Keywords:** reference intervals, hematology, clinical pathology, wild birds, wildlife medicine

## Abstract

**Objective:**

Reference intervals for hematologic and clinical chemistry values are useful when diagnosing a pathologic condition in animals. This study establishes relevant reference intervals for six species of wild birds that are frequently rescued at wildlife rescue centers in the Republic of Korea.

**Methods:**

Forty-two Eurasian eagle owls (*Bubo bubo*), 34 Oriental turtle doves (*Streptopelia orientalis*), 73 domestic pigeons (*Columba livia domestica*), 27 brown hawk-owls (*Ninox scutulata*), 76 common kestrels (*Falco tinnunculus*), and 25 Eurasian magpies (*Pica pica*) were included in this study. Only released birds were included because they were judged to be clinically healthy through physical examinations, blood examinations, radiographic examinations, and flight evaluations. The reference intervals were set according to the American Society for Veterinary Clinical Pathology guideline, and if there were fewer than 20 birds, the reference intervals were set between the 2.5th percentile and the 97.5th percentile. One-way ANOVA was performed to compare hematologic and clinical chemistry parameters among species.

**Results:**

The total protein levels in carnivorous birds (Eurasian eagle owl, brown hawk-owl, and common kestrel) were significantly higher than those in omnivorous birds (Oriental turtle dove and domestic pigeon). The common kestrel exhibited significantly lower white blood cell counts and heterophil counts than other species. The Eurasian magpie had significantly higher eosinophils than other species.

**Conclusion:**

This study provides reference intervals for wild birds often rescued at wildlife rescue centers in Korea. It is expected that these reference intervals will be used as important data in diagnosing diseases in rescued wild birds.

## 1 Introduction

Hematologic and clinical chemistry reference intervals represent the typical range expected in healthy individuals. Knowing such intervals allows clinicians to monitor an individual’s health status and easily identify individuals who might be affected by illness or experiencing deteriorating health (1). In the wild animals, hematologic and clinical chemistry reference intervals can be valuable, as well as for humans and domesticated animals (2). The various components within blood and their ranges reflect physiological and pathological changes, such as exposure to toxins and infectious disease, and can aid in the detection of environmental shifts and the effects of external factors. Hematological studies of wild birds contribute to understanding a species’ physiological characteristics and are also essential for monitoring and conserving ecosystem health (3, 4).

In Korea, 17 wildlife rescue centers are operated with government support, and approximately 20,000 wild animals are rescued annually across the country. Seventy percent of all rescued animals are birds, and they are rescued and treated because they cannot fly. In the process of diagnosing the reason for their inability to fly, a blood analysis can help to determine whether rescued wild birds have traumatic or physiological abnormalities in their internal organs and also whether they are in a condition suitable for surgery or anesthesia. In particular, wild animals have a tendency to hide clinical symptoms, making it challenging to diagnose disease during physical examination and provide appropriate medical procedures. A blood analysis can be helpful in this regard by facilitating the early diagnosis of disease. However, the research needed to establish hematology and clinical chemistry reference intervals for wildlife is scarce, and even when data are available, they often pertain to captive wildlife where they are fed a fixed diet and raised in restricted environments (5, 6). Research on hematological and clinical chemistry reference intervals for wild bird species native to the Republic of Korea is very limited (7, 8). This study aims to establish hematologic and clinical chemistry reference intervals for six species (Eurasian eagle owl [*Bubo bubo*], common kestrel [*Falco tinnunculus*], Oriental turtle dove [*Streptopelia orientalis*], domestic pigeon [*Columba livia domestica*], Eurasian magpie [*Pica pica*] and Brown hawk owl [*Ninox scutulata*]) of wild birds most frequently rescued from rescue centers in Korea, providing critical diagnostic tools for wildlife rehabilitation and conservation. Among the six species, the Eurasian eagle owl, common kestrel, and brown hawk owl are bird species designated as natural monuments in Korea.

## 2 Materials and methods

### 2.1 Sample collection

Among all birds that were rescued from 2017 to 2023 in Jeonbuk province, Republic of Korea, the medical records of clinically healthy birds were retrospectively collected and used for this study. To confirm that the birds were clinically healthy, all birds underwent at least one physical examination, blood examination (complete blood count [CBC], clinical chemistry, and electrolyte and blood gas analyses), radiographic examination, and flight evaluation. In addition to changes in white blood cells in the CBC, a blood film examination was conducted to evaluate the presence of inflammation in the early stage by determining whether toxic changes in heterophils appeared. If no abnormalities were found in any of the tests, the birds were judged to be clinically healthy. If a bird had any issues, it was included in this study only if additional tests (fecal examination, ophthalmic examination, ultrasound, computed tomography, magnetic resonance imaging, or molecular analysis) indicated that it had fully recovered. Additionally, the following information in the medical records was examined and analyzed: age, sex (if possible), reason for rescue, rescue area, and rescue date.

For blood tests, peripheral blood was collected from the brachial wing vein or jugular vein using a 23-G or 26-G needle. The collected blood was put into ethylene-diamine-tetraacetic acid (EDTA)- and heparin-treated tubes. CBC and clinical chemistry profile tests were completed within 3 h after blood collection by experienced veterinarians or clinical pathology technicians. The remaining blood was stored at −20°C and used if additional testing was needed.

### 2.2 Hematological profiles

Blood samples collected in EDTA tubes were primarily used. In cases in which the bird’s blood samples exhibited characteristics of hemolysis in EDTA, we used blood samples collected in heparin-treated tubes. CBC was conducted manually to determine the packed cell volume (PCV), hemoglobin (Hb) concentration, and concentrations of red blood cells (RBCs) and white blood cells (WBCs). A blood smear was prepared and stained with Diff-Quik (Sysmex, Kobe, Japan), and then a differential count was additionally performed by counting 100 WBCs under a light microscope. The mean corpuscular volume (MCV), mean corpuscular Hb (MCH), and mean corpuscular Hb concentration (MCHC) were calculated using the following formulae.


$$MCV=\frac{PCV}{RBC}\times 10$$

$$MCH=\frac{Hb}{RBC}\times 10$$

$$\mathrm{MCHC}=\frac{Hb}{PCV}\times 100$$

### 2.3 Clinical chemistry profiles

Clinical chemistry profiles were developed using blood samples collected in heparin-treated tubes and automated analyzers (Vetscan VS2, Abaxis Inc., Union City, California, USA). The following items were analyzed: albumin, aspartate aminotransferase (AST), bile acid, calcium, creatine kinase (CK), glucose, globulin, phosphorus, potassium, sodium, total protein, and uric acid.

### 2.4 Statistical analysis

All procedures for establishing reference intervals were performed in accordance with the American Society for Veterinary Clinical Pathology guidelines (9). In accordance with the guidelines, data from 20 or more individuals per species were included in this study.

A normal distribution evaluation and one-way ANOVA were performed using SPSS 20 statistical software (IBM SPSS Statistics, Chicago, Illinois, USA). Excel 2016 (Microsoft Corp., Redmond, WA, USA) with macroinstructions from Reference Value Advisor was used to set the reference intervals (10).

When data were collected from more than 20 individuals per species, the normal distribution of the data was evaluated using the Kolmogorov–Smirnov test (sample size <50) or Shapiro–Wilk test (sample size >50) (11).

In Gaussian data, reference intervals were determined using parametric methods. In non-Gaussian data, either a parametric method after Box-Cox transformation or a nonparametric method was chosen, as recommended in Reference Value Advisor.

When the sample size was less than 20, the mean, standard deviation, minimum, and maximum were calculated in SPSS 20 (IBM SPSS Statistics). The reference intervals were set between the 2.5th percentile and the 97.5th percentile (12). The normal distribution of the data was evaluated using the Kolmogorov–Smirnov test (11).

One-way ANOVA was performed to compare hematologic and clinical chemistry parameters among species. A *p* value <0.05 was considered to indicate statistical significance, and Tukey’s HSD post-hoc test was performed to identify which species pairs exhibited significant differences.

## 3 Results

### 3.1 Animals

Only birds deemed to be clinically healthy were included in the results. Data from 42 Eurasian eagle owls (*Bubo bubo*), 34 Oriental turtle doves (*Streptopelia orientalis*), 73 domestic pigeons (*Columba livia domestica*), 27 brown hawk-owls (*Ninox scutulata*), 76 common kestrels (*Falco tinnunculus*), and 25 Eurasian magpies (*Pica pica*) were evaluated in this study.

### 3.2 Comparison of hematologic and clinical chemistry parameters among the six species

One-way ANOVA was performed to compare the hematologic and clinical chemistry parameters of the six species. Among the hematologic parameters, the lymphocyte percentage (*p* = 0.22), basophil percentage (*p* = 0.85), lymphocytes (*p* = 0.06), and basophils (*p* = 0.85) did not differ significantly among the species. In the clinical chemistry, albumin (*p* = 0.60), glucose (*p* = 0.58), and bile acid (*p* = 0.15) did not differ significantly among species. The hematologic results are shown in Table 1, and the clinical chemistry results are shown in Table 2.

**Table 1:** One-way ANOVA comparing hematologic parameters among six species.

Parameter (SI units)		df	MS	F	*p* value
WBCs (10^3^/μL)	1^a^	5	325.96	7.57	0.00
	2^b^	263	43.06		
Heterophils per (%)	1^a^	5	871.33	2.54	0.03
	2^b^	248	343.44		
Lymphocytes per (%)	1^a^	5	265.49	1.41	0.22
	2^b^	248	188.04		
Monocytes per (%)	1^a^	5	255.34	2.65	0.24
	2^b^	248	96.46		
Eosinophils per (%)	1^a^	5	1680.80	30.03	0.00
	2^b^	246	55.96		
Basophils per (%)	1^a^	5	0.12	0.39	0.85
	2^b^	248	0.04		
Heterophils (10^3^/μL)	1^a^	5	161.47	5.98	0.00
	2^b^	248	26.97		
Lymphocytes (10^3^/μL)	1^a^	5	7.52	2.13	0.06
	2^b^	246	3.54		
Monocytes (10^3^/μL)	1^a^	5	8.03	3.62	0.00
	2^b^	247	2.22		
Eosinophils (10^3^/μL)	1^a^	5	30.15	5.65	0.00
	2^b^	247	5.34		
Basophils (10^3^/μL)	1^a^	5	0.00	1.00	0.42
	2^b^	260	0.00		
RBCs^*^ (10^6^/μL)	1^a^	5	5.06	8.55	0.00
	2^b^	269	0.59		
Packed cell volume (%)	1^a^	5	286.76	10.28	0.00
	2^b^	252	27.89		
Hb^*^ concentration (g/dL)	1^a^	5	45.77	4.87	0.00
	2^b^	257	9.41		
Mean corpuscular volume^*^ (fL)	1^a^	5	10476.93	7.89	0.00
	2^b^	257	1328.48		
Mean corpuscular hemoglobin^*^ (pg)	1^a^	5	4817.05	10.95	0.00
	2^b^	251	439.89		
Mean corpuscular hemoglobin concentration^*^ (g/㎗)	1^a^	5	390.06	6.75	0.00
	2^b^	251	57.76		

WBC, white blood cell; RBC, red blood cell; Hb, hemoglobin; MCV, mean corpuscular volume; MCH, mean corpuscular hemoglobin; MCHC, mean corpuscular hemoglobin concentration; df, degrees of freedom; MS, mean square 1^a^ interspecies; 2^b^ intraspecies.

**Table 2:** One-way ANOVA comparing clinical chemistry parameters among six species.

Parameter (SI units)		df	MS	F	*p* value
Total protein (g/㎗)	1^a^	5	4.83	12.23	0.00
	2^b^	157	0.39		
Potassium (mmol/L)	1^a^	5	10.52	3.99	0.00
	2^b^	158	2.64		
Calcium (㎎/㎗)	1^a^	5	3.01	4.15	0.00
	2^b^	160	0.73		
Phosphorus (㎎/㎗)	1^a^	5	17.69	5.04	0.00
	2^b^	159	3.51		
Sodium (mmol/L)	1^a^	5	537.80	20.18	0.00
	2^b^	161	26.65		
Albumin (g/㎗)	1^a^	5	0.21	0.73	0.06
	2^b^	157	0.29		
Globulin (g/㎗)	1^a^	5	4.62	16.95	0.00
	2^b^	152	00.27		
AST (IU/L)	1^a^	5	1440584.98	8.54	0.00
	2^b^	159	168754.22		
Glucose (mg/dL)	1^a^	5	3178.34	0.76	0.58
	2^b^	161	4185.57		
CK (IU/L)	1^a^	5	10432425.41	7.67	0.00
	2^b^	159	1360276.87		
Uric acid (mg/dL)	1^a^	5	215.75	8.30	0.00
	2^b^	157	25.99		
Bile acid (μmol/L)	1^a^	5	280.51	1.67	0.15
	2^b^	159	168.45		

AST, aspartate aminotransferase; CK, creatine kinase; df, degrees of freedom; MS, mean square 1^a^ interspecies; 2^b^ intraspecies.

### 3.3 Hematologic and clinical chemistry reference intervals in Eurasian eagle owls (*Bubo bubo*)

The hematologic reference intervals for Eurasian eagle owls reflect data from 42 individuals, and the clinical chemistry reference intervals reflect data from 21 animals. Among the parameters, eosinophils (eosinophil percentage), MCV, MCH, AST, CK, and bile acid were found to be non-Gaussian, and the rest of the parameters were confirmed to be Gaussian.

The RBC count of the Eurasian eagle-owl did not differ significantly from that of the brown hawk-owl and Eurasian magpie, but it was significantly lower than in the Oriental turtle dove, domestic pigeon, and common kestrel. On the other hand, its MCV was significantly higher than in any of the other species. The MCH did not differ significantly from the brown hawk-owl, but it was higher than in the other species. Similarly, MCHC did not differ significantly from the brown hawk-owl or common kestrel, but it was higher than in the other species. Calcium was significantly higher than in the domestic pigeon and common kestrel, and phosphorus was significantly higher than in the brown hawk-owl and Eurasian magpie. Total protein was significantly higher than in the domestic pigeon and Oriental turtle dove. The hematologic reference intervals and clinical chemistry reference intervals for Eurasian eagle owls are shown in Tables 3, 4, respectively.

**Table 3:** Hematologic reference intervals for Eurasian eagle owls.

Parameter (SI units)	N	Mean	SD	Min	Max	RI	L	U	D
WBCs (10^3^/μL)	42	11.6	4.6	4.0	27.0	4.0–26.5	4.0–6.0	18.0–27.0	G
Heterophils per (%)	37	69.4	17.4	22.0	95.0	15.7–95.8	ND-40.8	91.4–100.2	G
Lymphocytes per (%)	37	15.8	12.9	1.0	55.0	0.0–49.2	0.0–1.1	38.5–61.7	G
Monocytes per (%)	37	13.3	9.1	0	34.0	0–32.8	0.0	26.2–39.4	G
Eosinophils per (%)	37	1.7	3.6	0	16.0	0–9.1	0.0	4.5–12.3	NG
Basophils per (%)	37	0.3	0.2	0	1.0	0.0	0.0	0.0	G
Heterophils (10^3^/μL)	37	8.00	4.2	2.3	23.8	2.4–19.2	1.9–3.3	15.5–23.6	G
Lymphocytes (10^3^/μL)	37	1.9	1.7	0.1	6.9	0.0–5.3	0.0	4.0–6.5	G
Monocytes (10^3^/μL)	37	1.5	1.0	0	4.3	0–3.5	0.0	3.0–4.0	G
Eosinophils (10^3^/μL)	37	0.5	1.7	0	10.8	0–2.7	0.0	0.7–5.1	NG
Basophils (10^3^/μL)	37	0.0	0.2	0	1.0	0–0.3	0.0	0.0–0.6	G
RBCs (10^6^/μL)	42	2.4	0.7	1.2	3.8	1.3–3.8	1.2–1.5	3.4–3.8	G
PCV (%)	41	35.7	4.00	30.0	45.0	30.0–45.0	30.0	42.9–45.0	G
Hb concentration (g/dL)	40	16.4	3.7	10.6	25.5	10.6–25.4	10.6–11.9	23.0–25.5	G
Mean corpuscular volume (fL)	41	165.4	54.8	92.0	275.4	92.3–275.3	92.0–102.3	249.4–275.4	NG
Mean corpuscular hemoglobin (pg)	40	78.4	34.2	31.6	174.4	31.7–173.4	31.6–40.6	128.5–174.4	NG
Mean corpuscular hemoglobin concentration (g/㎗)	39	46.6	9.5	27.1	69.8	27.0–66.1	24.1–30.9	59.9–71.0	G

N, number (sample size); WBC, white blood cell; RBC, red blood cell; PCV, packed cell volume; Hb, hemoglobin; MCV, mean corpuscular volume; MCH, mean corpuscular hemoglobin; MCHC, mean corpuscular hemoglobin concentration; SD, standard deviation; Min, minimum; Max, maximum; RI, reference interval; L, 90% confidence interval for lower limit; U, 90% confidence interval for upper limit; ND, not determined; D, distribution; G, Gaussian; NG, non-Gaussian.

**Table 4:** Clinical chemistry reference intervals for Eurasian eagle owls.

Parameter (SI units)	N	Mean	SD	Min	Max	RI	L	U	D
Total protein (g/㎗)	21	3.8	0.7	2.5	4.9	2.4–5.2	2.0–2.8	4.8–5.6	G
Potassium (mmol/L)	20	4.1	1.0	2.1	5.7	1.9–6.3	1.3–2.7	5.6–7.0	G
Calcium (㎎/㎗)	21	10.0	1.3	5.8	11.6	7.3–12.7	5.8–9.1	11.6–13.4	G
Phosphorus (㎎/㎗)	20	5.3	2.7	2.2	14.7	0.00–11.1	0–1.9	7.4–14.6	G
Sodium (mmol/L)	21	153.2	4.5	146.0	161.0	143.6–162.8	140.5–146.4	159.7–165.9	G
Albumin (g/㎗)	21	2.1	0.6	1.0	3.1	0.9–3.3	0.6–1.3	2.9–3.6	G
Globulin (g/㎗)	21	1.7	0.5	0.6	2.4	0.6–2.7	0.3–0.9	2.4–3.0	G
AST (IU/L)	21	494.8	634.9	19.0	3033.0	31.6–7505.9	20.8–96.4	1120.6–44561.2	NG
Glucose (mg/dL)	21	350.1	67.6	228.0	568.0	203.4–488.2	137.5–275.0	425.8–544.4	G
CK (IU/L)	20	1167.8	1148.6	306.0	5000.0	536.6–780.8	506.9–576.3	712.7–842.1	NG
Uric acid (mg/dL)	20	5.9	3.6	1.2	15.6	0.0–12.8	0.0–0.2	9.1–15.8	G
Bile acid (μmol/L)	20	32.4	7.6	0	35.0	16.0–48.7	2.2–34.0	34.0–55.7	NG

N, number (sample size); AST, aspartate aminotransferase; CK, creatine kinase; SD, standard deviation; Min, minimum; Max, maximum; RI, reference interval; L, 90% confidence interval for lower limit; U, 90% confidence interval for upper limit; D, distribution; G, Gaussian; NG, non-Gaussian.

### 3.4 Hematologic and clinical chemistry reference intervals in Oriental turtle doves (*Streptopelia orientalis*)

The hematologic reference intervals for Oriental turtle doves reflect data from 34 individuals, whereas the clinical chemistry reference intervals reflect data from 18 animals. Among the parameters, the heterophil percentage, lymphocyte percentage, monocytes, RBCs, PCV, MCV, MCH, MCHC, total protein, phosphorus, albumin, globulin, glucose, and uric acid were found to be Gaussian, and the rest of the parameters were confirmed to be non-Gaussian.

Potassium and CK in the Oriental turtle dove were significantly higher than in the domestic pigeon. Total protein was lower than in the Eurasian eagle owl and brown hawk-owl. The hematologic reference intervals and clinical chemistry reference intervals for Oriental turtle doves are shown in Tables 5, 6, respectively.

**Table 5:** Hematologic reference intervals in Oriental turtle doves.

Parameter (SI units)	N	Mean	SD	Min	Max	RI	L	U	D
WBCs (10^3^/μL)	32	11.6	9.0	0.5	43.0	0.8–36.8	0.3–2.0	27.6–49.3	NG
Heterophils per (%)	31	69.7	17.2	27	94	34.0–105.4	25.2–43.0	95.8–114.-5	G
Lymphocytes per (%)	31	20.4	16.2	0	64	0–54.0	0	45.1–62.8	G
Monocytes per (%)	31	9.8	8	0	43	0–25.3	0	17.2–32.7	NG
Eosinophils per (%)	31	0.1	0.4	0	2	0–1.0	0	0.4–1.6	NG
Basophils per (%)	31	0	0	0	0	0	0	0	NG
Heterophils (10^3^/μL)	31	8.5	7.7	0.3	33.1	0.4–32.3	0.1–1.0	22.3–44.1	NG
Lymphocytes (10^3^/μL)	31	2	2.2	0	9.6	0.0–8.8	0.0–0.1	5.7–12.5	NG
Monocytes (10^3^/μL)	31	1.1	1	0	3.6	0.0–3.1	0	2.3–3.8	G
Eosinophils (10^3^/μL)	31	0.1	0.1	0	0.2	0.0–0.0	0	0.0–0.2	NG
Basophils (10^3^/μL)	31	0	0	0	0	0	0	0	NG
RBCs (10^6^/μL)	32	3.2	1	1.6	6.8	1.2–5.1	0.7–1.8	4.3–6.0	G
PCV (%)	34	38.2	6.3	29	50	25.2–51.1	21.9–28.4	47.9–54.3	G
Hb^*^ concentration (g/dL)	31	14.9	2.9	11	22.5	10.6–23.5	10.1–11.4	20.3–27.4	NG
Mean corpuscular volume (fL)	32	127.3	30.9	63.6	206	63.4–191.3	48.2–79.1	175.1–207.2	G
Mean corpuscular hemoglobin (pg)	31	50.6	15	23	102.9	19.5–81.6	11.9–27.7	73.5–89.4	G
Mean corpuscular hemoglobin concentration (g/㎗)	31	39.3	5.7	27.6	56.3	27.5–51.1	24.5–30.7	48.0–53.8	G

N, number (sample size); WBC, white blood cell; RBC, red blood cell; PCV, packed cell volume; Hb, hemoglobin; MCV, mean corpuscular volume; MCH, mean corpuscular hemoglobin; MCHC, mean corpuscular hemoglobin concentration; SD, standard deviation; Min, minimum; Max, maximum; RI, reference interval; L, 90% confidence interval for lower limit; U, 90% confidence interval for upper limit; ND, not determined; D, distribution; G, Gaussian; NG, non-Gaussian.

**Table 6:** Clinical chemistry reference intervals in Oriental turtle doves.

Parameter (SI units)	N	Mean	SD	Min	Max	RI	D
Total protein (g/㎗)	18	3	0.7	1.6	4.3	1.6–4.0	G
Potassium (mmol/L)	18	5.1	3.6	1.6	18	1.6–8.5	NG
Calcium (㎎/㎗)	18	9.3	1.1	8.4	12.7	8.4–10.5	NG
Phosphorus (㎎/㎗)	18	4.5	1.8	2.3	8.7	2.3–6.7	G
Sodium (mmol/L)	18	151.3	9	139	180	139.0–158.0	G
Albumin (g/㎗)	18	2.2	0.4	1.5	3	1.5–2.8	G
Globulin (g/㎗)	18	0.8	0.6	0	2	0.0–1.7	NG
AST (IU/L)	18	320.3	411.8	74	1,500	74.0–1277.0	NG
Glucose (mg/dL)	18	334.2	28.7	282	380	282.0–378.0	G
CK (IU/L)	18	2265.8	1509.5	797	5,000	797.0–4554.0	NG
Uric acid (mg/dL)	18	6.5	3	2.1	12.5	2.1–11.8	G
Bile acid (μmol/L)	18	39.6	22.3	4	94	4.0–75.0	NG

N, number (sample size); AST, aspartate aminotransferase; CK, creatine kinase; SD, standard deviation; RI, reference interval; G, Gaussian; NG, non-Gaussian.

### 3.5 Hematologic and clinical chemistry reference intervals in Domestic pigeons (*Columba livia domestica*)

The hematologic reference intervals for domestic pigeons reflect data from 73 individuals, whereas the clinical chemistry reference intervals reflect data from 51 animals. Among the parameters, heterophil percentage, lymphocyte percentage, monocyte percentage, RBCs, Hb, total protein, calcium, albumin, and uric acid were found to be Gaussian, and the rest of the parameters were confirmed to be non-Gaussian.

The PCV of the domestic pigeon was significantly higher than in the Eurasian eagle owl and common kestrel. The Hb concentration was also significantly higher than in the common kestrel. Sodium was significantly lower than in all the other species. Total protein was lower than in the brown hawk-owl and common kestrel, and AST was lower than in the brown hawk-owl and Eurasian magpie. Uric acid was significantly lower than in the common kestrel. The hematologic reference intervals and clinical chemistry reference intervals for domestic pigeons are shown in Tables 7, 8, respectively.

**Table 7:** Hematologic reference intervals in domestic pigeons.

Parameter (SI units)	N	Mean	SD	Min	Max	RI	L	U	D
WBCs (10^3^/μL)	70	9.2	6.6	1.5	33.0	1.5–30.3	1.5–2.5	21.6–33.0	NG
Heterophils per (%)	67	63.9	15.1	30.0	91.0	32.1–89.6	30.0–36.8	88.0–91.0	G
Lymphocytes per (%)	67	20.8	11.7	1.0	60.0	3.1–55.1	1.0–5.7	41.1–60.0	G
Monocytes per (%)	67	15.7	10.7	0	60.0	0.7–49.5	0.0–3.1	34.2–60.0	G
Eosinophils per (%)	67	0.3	0.7	0	3.0	0–3.0	0.0	1.3–3.0	NG
Basophils per (%)	67	0.0	0.3	0	2.0	0–1.3	0.0	2.0	NG
Heterophils (10^3^/μL)	67	5.6	4.2	0	19.8	0.7–16.9	0.0–1.3	13.2–19.8	NG
Lymphocytes (10^3^/μL)	67	1.9	2.6	0	17.7	0.1–12.7	0.0–0.3	4.9–17.7	NG
Monocytes (10^3^/μL)	67	1.5	2.3	0	1.7	0–8.8	0.0–0.1	4.1–17.7	NG
Eosinophils (10^3^/μL)	67	0.2	0.7	0	0.5	0–0.4	0.0	0.1–0.5	NG
Basophils (10^3^/μL)	67	0.0	0.0	0	0.3	0–0.2	0.0	0.0–0.3	NG
RBCs (10^6^/μL)	68	3.3	0.9	1.9	5.1	1.9–5.0	1.9–2.1	4.9–5.1	G
PCV (%)	72	40.7	5.8	25.0	53.0	26.7–52.2	25.0–30.0	49.2–53.0	NG
Hb concentration (g/dL)	65	16.3	2.9	8.7	23.5	10.2–23.5	8.7–12.0	21.0–23.5	G
Mean corpuscular volume (fL)	67	133.2	34.3	75.8	232.2	81.4–223.6	75.8–88.2	187.0–232.2	NG
Mean corpuscular hemoglobin (pg)	65	53.4	18.9	23.3	102.1	26.5–96.8	22.3–31.6	86.2–102.1	NG
Mean corpuscular hemoglobin concentration (g/㎗)	65	40.1	6.9	17.4	59.9	21.9–57.3	17.4–31.6	52.1–59.9	NG

N, number (sample size); WBC, white blood cell; RBC, red blood cell; PCV, packed cell volume; Hb, hemoglobin; MCV, mean corpuscular volume; MCH, mean corpuscular hemoglobin; MCHC, mean corpuscular hemoglobin concentration; SD, standard deviation; Min, minimum; Max, maximum; RI, reference interval; L, 90% confidence interval for lower limit; U, 90% confidence interval for upper limit; D, distribution; G, Gaussian; NG, non-Gaussian.

**Table 8:** Clinical chemistry reference intervals in domestic pigeons.

Parameter (SI units)	N	Mean	SD	Min	Max	RI	L	U	D
Total protein (g/㎗)	46	2.7	0.6	1.7	4.7	1.7–4.5	1.7–1.9	3.7–4.7	G
Potassium (mmol/L)	*49*	3.4	1.4	1.4	8.3	1.4–7.9	1.4–1.5	5.1–8.3	NG
Calcium (㎎/㎗)	*49*	9	0.8	5.4	10.6	6.00–10.5	5.4–8.2	10.1–10.6	G
Phosphorus (㎎/㎗)	*49*	4.2	2.1	0.5	12.6	0.6–11.7	0.5–1.5	6.8–12.6	NG
Sodium (mmol/L)	*50*	144.1	4.9	130	157	131.1–155.6	130.0–136.0	150.7–157.0	NG
Albumin (g/㎗)	*46*	2.3	0.5	0.9	3.4	1.0–3.4	0.9–1.4	3.1–3.4	G
Globulin (g/㎗)	40	0.5	0.6	0	2.5	0–2.5	0	1.0–2.5	NG
AST (IU/L)	48	180.5	334.8	33	2,210	36.6–1947.7	33.0–51.0	331.2–2210.0	NG
Glucose (mg/dL)	50	333.9	62.4	244	701	245.1–629.5	244.0–268.4	386.0–701.0	NG
CK (IU/L)	50	833.2	687.8	197	3,672	215.2–3611.0	197.0–306.9	1564.0–3672.0	NG
Uric acid (mg/dL)	49	3.4	1.9	0.7	7.5	0.7–7.4	0.7–1.0	6.4–7.5	G
Bile acid (μmol/L)	49	30.2	14.3	0	73	0–69.0	0.0–3.0	51.8–73.0	NG

N, number (sample size); AST, aspartate aminotransferase; CK, creatine kinase; SD, standard deviation; Min, minimum; Max, maximum; RI, reference interval; L, 90% confidence interval for lower limit; U, 90% confidence interval for upper limit; D, distribution; G, Gaussian; NG, non-Gaussian.

### 3.6 Hematologic and clinical chemistry reference intervals in Brown hawk-owls (*Ninox scutulata*)

The hematologic reference intervals for brown hawk-owls reflect data from 27 individuals, whereas the clinical chemistry reference interval reflects data from 21 animals. Among the parameters, heterophil percentage, PCV, Hb, MCV, MCH, total protein, potassium, calcium, phosphorus, glucose, CK, and uric acid were found to be Gaussian, and the rest of the parameters were confirmed to be non-Gaussian.

Total protein was significantly higher than in domestic pigeons and Oriental turtle doves. CK was significantly higher than in Eurasian eagle owls and domestic pigeons. The hematologic reference intervals and clinical chemistry reference intervals for brown hawk-owls are shown in Tables 9, 10, respectively.

**Table 9:** Hematologic reference intervals in brown hawk-owls.

Parameter (SI units)	N	Mean	SD	Min	Max	RI	L	U	D
WBCs (10^3^/μL)	27	12.1	10.2	1.5	49	2.1–49.4	1.6–3.0	28.1–81.8	NG
Heterophils per (%)	27	63.2	16.6	32	92	28.4–97.9	19.5–37.6	88.0–107.5	G
Lymphocytes per (%)	27	20.1	12.6	4	60	3.2–50.9	ND-5.1	38.7–62.5	NG
Monocytes per (%)	27	14.9	13	0	49	0.1–47.8	ND-2.0	36.1–59.7	NG
Eosinophils per (%)	27	1.9	3.9	0	18	0.1–47.8	ND-2.2	35.8–59.4	NG
Basophils per (%)	27	0	0	0	0	0	0	0	NG
Heterophils (10^3^/μL)	27	8.1	8.9	0.9	38.7	1.1–47.6	0.9–1.5	21.6–99.3	NG
Lymphocytes (10^3^/μL)	27	1.8	1.4	0.2	6	0.2–6.6	0–0.5	3.9–18.1	NG
Monocytes (10^3^/μL)	27	1.4	1.7	0	8.3	4.0–10.3	3.7–4.2	7.5–22.0	NG
Eosinophils (10^3^/μL)	27	1.3	5.6	0	29	0.0–13.0	0	0.5–22.8	NG
Basophils (10^3^/μL)	27	0	0	0	0	0	0	0	NG
RBCs (10^6^/μL)	27	2.7	0.6	1.9	4	1.7–4.4	1.6–1.9	3.7–5.2	NG
PCV (%)	27	38	5	30	48	27.4–48.5	24.6–30.5	45.7–51.4	G
Hb^*^ concentration (g/dL)	27	16.8	3.7	11	24	9.1–24.5	7.0–11.3	22.3–26.7	G
Mean corpuscular volume (fL)	27	148	35	99	243.4	93.5–243.1	84.4–103.9	208.3–292.0	G
Mean corpuscular hemoglobin (pg)	27	65.9	21.3	32.6	125.8	32.8–120.6	27.9–39.8	100.2–141.4	G
Mean corpuscular hemoglobin concentration (g/㎗)	27	44.3	8.4	30.5	59.7	29.7–64.9	26.9–32.4	58.2–72.4	NG

N, number (sample size); WBC, white blood cell; RBC, red blood cell; PCV, packed cell volume; Hb, hemoglobin; MCV, mean corpuscular volume; MCH, mean corpuscular hemoglobin; MCHC, mean corpuscular hemoglobin concentration; SD, standard deviation; Min, minimum; Max, maximum; RI, reference interval; L, 90% confidence interval for lower limit; U, 90% confidence interval for upper limit; ND, not determined; D, distribution; G, Gaussian; NG, non-Gaussian.

**Table 10:** Clinical chemistry reference intervals in brown hawk-owls.

Parameter (SI units)	N	Mean	SD	Min	Max	RI	L	U	D
Total protein (g/㎗)	21	3.7	0.4	2.6	4.5	2.8–4.7	2.5–3.0	4.4–5.0	G
Potassium (mmol/L)	21	3.6	0.8	2.1	5.2	1.8–5.4	1.3–2.5	4.8–5.9	G
Calcium (㎎/㎗)	21	9.2	0.6	8.2	10.4	8.0–10.4	7.6–8.4	10.0–10.8	G
Phosphorus (㎎/㎗)	21	3.2	1.2	0.8	5.0	0.6–5.8	0.0–1.4	5.1–6.6	G
Sodium (mmol/L)	21	152.4	4.8	142.0	165.0	143.1–163.5	140.7–145.8	159.3–168.5	NG
Albumin (g/㎗)	21	3.6	0.7	1.1	3.4	0.9–3.5	0.4–1.4	3.1–3.8	NG
Globulin (g/㎗)	21	1.4	0.5	0.3	2.3	0.0–2.5	ND-0.6	2.2–2.8	NG
AST (IU/L)	21	567.3	578.1	43.0	2000.0	0.0–1615.7	ND	906.6–2140.0	NG
Glucose (mg/dL)	21	337.5	72.8	212.0	547.0	221.8–534.8	199.2–248.4	461.9–637.1	G
CK (IU/L)	21	2398.1	1514.0	196.0	6196.0	185.1–6671.8	32.4–588.8	4863.0–8502.0	G
Uric acid (mg/dL)	21	6.1	3.1	1.0	12.5	0.5–13.7	ND-2.1	11.5–16.3	NG
Bile acid (μmol/L)	21	35.1	5.0	34.0	57.0	24.4–45.8	19.7–34.0	34.0–54.9	NG

N, number (sample size); AST, aspartate aminotransferase; CK, creatine kinase; SD, standard deviation; Min, minimum; Max, maximum; RI, reference interval; L, 90% confidence interval for lower limit; U, 90% confidence interval for upper limit; D, distribution; G, Gaussian; NG, non-Gaussian.

### 3.7 Hematologic and clinical chemistry reference intervals in Common kestrels (*Falco tinnunculus*)

The hematologic reference intervals for the common kestrel reflect data from 76 individuals, and the clinical chemistry reference intervals reflect data from 46 animals. Among the parameters, lymphocyte percentage, RBCs, PCV, Hb, MCHC, total protein, potassium, calcium, sodium, albumin, and phosphorus were found to be Gaussian, and the rest of the parameters were confirmed to be non-Gaussian.

WBCs and heterophils were significantly lower than in all the other species. The hematologic reference intervals and clinical chemistry reference intervals for the common kestrel are shown in Tables 11, 12, respectively.

**Table 11:** Hematologic reference intervals in common kestrels.

Parameter (SI units)	N	Mean	SD	Min	Max	RI	L	U	D
WBCs (10^3^/μL)	74	5.6	4.0	0.5	18.5	0.5–16.3	0.5–1.4	13.5–18.5	NG
Heterophils per (%)	69	65.6	22.0	7.0	99.0	7.8–96.8	7.0–32.8	90.5–99.0	NG
Lymphocytes per (%)	69	21.8	16.4	0.0	78.0	0.8–72.0	0.0–1.8	49.3–78.0	G
Monocytes per (%)	69	10.7	8.2	0.0	31.0	0.0–31.0	0.0–1.0	26.0–31.0	NG
Eosinophils per (%)	69	0.3	0.8	0.0	4.0	0.0–4.0	0.0	1.5–4.0	NG
Basophils per (%)	69	0.0	0.2	0.0	2.0	0.0–0.6	0.0	0.0–2.0	NG
Heterophils (10^3^/μL)	69	3.9	3.3	0.2	14.8	0.2–13.9	0.2–0.4	10.4–14.8	NG
Lymphocytes (10^3^/μL)	69	1.0	1.1	0.0	5.1	0.0–4.6	0.0–0.1	3.2–5.1	NG
Monocytes (10^3^/μL)	69	0.5	0.6	0.0	3.7	0.0–2.8	0.0	1.6–3.7	NG
Eosinophils (10^3^/μL)	69	0.0	0.1	0.0	0.3	0.0–0.2	0.0	0.1–0.3	NG
Basophils (10^3^/μL)	69	0.0	0.0	0.0	0.0	0.0	0.0	0.0	NG
RBCs (10^6^/μL)	74	2.9	0.6	1.2	4.4	1.6–4.2	1.2–1.7	3.7–4.4	G
PCV (%)	76	34.9	5.2	20.0	45.0	24.5–45.3	20.0–24.9	43.0–45.0	G
Hb concentration (g/dL)	71	14.5	2.9	7.7	21.4	8.9–20.3	7.7–10.3	19.8–21.4	G
Mean corpuscular volume (fL)	73	124.6	26.3	82.9	223.9	87.9–203.6	82.9–94.4	178.3–223.9	NG
Mean corpuscular hemoglobin (pg)	71	52.8	15.9	29.6	98.1	31.4–92.1	29.6–34.3	85.8–98.1	NG
Mean corpuscular hemoglobin concentration (g/㎗)	71	42.0	8.1	27.7	69.2	30.3–62.8	27.7–32.1	58.4–61.2	G

N, number (sample size); WBC, white blood cell; RBC, red blood cell; PCV, packed cell volume; Hb, hemoglobin; MCV, mean corpuscular volume; MCH, mean corpuscular hemoglobin; MCHC, mean corpuscular hemoglobin concentration; SD, standard deviation; Min, minimum; Max, maximum; RI, reference interval; L, 90% confidence interval for lower limit; U, 90% confidence interval for upper limit; D, distribution; G, Gaussian; NG, non-Gaussian.

**Table 12:** Clinical chemistry reference intervals in common kestrels.

Parameter (SI units)	N	Mean	SD	Min	Max	RI	L	U	D
Total protein (g/㎗)	46	3.2	0.7	1.5	4.9	1.6–4.9	1.5–2.3	4.1–4.9	G
Potassium (mmol/L)	45	4.5	1.3	1.4	9.0	1.5–8.6	1.4–2.6	6.1–9.0	G
Calcium (㎎/㎗)	46	9.2	0.7	7.5	10.4	7.5–10.4	7.5–7.9	10.1–10.4	NG
Phosphorus (㎎/㎗)	46	3.1	1.5	0.0	6.3	0.0–6.2	0–0.9	5.2–6.3	G
Sodium (mmol/L)	46	151.0	4.2	140.0	161.0	140.9–160.7	140.0–146.0	158.0–161.0	G
Albumin (g/㎗)	46	2.3	0.6	0.9	3.5	0.9–3.5	0.9–1.4	3.2–3.5	G
Globulin (g/㎗)	46	1.0	0.5	0.0	2.3	0.0–2.3	0.0–0.3	2.0–2.3	NG
AST (IU/L)	46	117.4	162.4	34.0	857.0	34.5–853.3	34.0–46.0	198.2–857.0	NG
Glucose (mg/dL)	46	342.3	43.1	229.0	441.0	235.5–440.1	229.0–275.7	405.2–441.0	NG
CK (IU/L)	45	1563.3	861.6	429.0	5000.0	482.6–4838.5	429.0–808.2	2548.1–5000.0	NG
Uric acid (mg/dL)	45	10.1	8.4	1.8	50.0	1.8–46.0	1.8–2.2	21.6–50.0	NG
Bile acid (μmol/L)	44	31.9	11.5	0.0	64.0	0.7–27.3	0.0–3.4	44.8–64.0	NG

N, number (sample size); AST, aspartate aminotransferase; CK, creatine kinase; SD, standard deviation; Min, minimum; Max, maximum; RI, reference interval; L, 90% confidence interval for lower limit; U, 90% confidence interval for upper limit; D, distribution; G, Gaussian; NG, non-Gaussian.

### 3.8 Hematologic and clinical chemistry reference intervals in Eurasian magpies (*Pica pica*)

The hematologic reference intervals for Eurasian magpies reflect data from 25 individuals, and the clinical chemistry reference intervals reflect data from 11 animals. Among the parameters, monocytes, globulin, CK, and bile acid were found to non-Gaussian, and the rest of the parameters were confirmed to be Gaussian. The Eurasian magpie was characterized by significantly higher eosinophils than found in the other species. The hematologic reference intervals and clinical chemistry reference intervals for Eurasian magpies are shown in Tables 13, 14, respectively.

**Table 13:** Hematologic reference intervals in Eurasian magpies.

Parameter (SI units)	N	Mean	SD	Min	Max	RI	L	U	D
WBCs (10^3^/μL)	23	10.6	6.8	0.5	24.5	0–25.0	0–0.1	20.8–29.0	G
Heterophils per (%)	23	54.1	21.5	20.0	94.0	8.5–99.7	0–22.5	86.8–113.2	G
Lymphocytes per (%)	23	15.7	8.1	3.0	37.0	0–32.9	0–3.5	28.3–38.7	G
Monocytes per (%)	23	12.7	10.7	0.0	35.0	0.0–35.2	0.0	28.0–41.5	G
Eosinophils per (%)	23	20.6	24.2	0.0	60.0	0.0–71.9	0	54.5–83.5	G
Basophils per (%)	23	0.0	0.0	0.0	0.0	0.0	0.0	0.0	G
Heterophils (10^3^/μL)	23	5.1	3.7	0.2	17.4	0.4–15.3	0.1–1.1	10.7–20.5	G
Lymphocytes (10^3^/μL)	23	1.6	1.5	0.1	6.9	0.1–6.5	0.1–0.2	4.1–9.8	G
Monocytes (10^3^/μL)	23	1.1	1.2	0.0	3.8	0.0–5.8	0.0	3.1–8.4	NG
Eosinophils (10^3^/μL)	23	2.5	4.3	0.0	13.7	0.0–11.6	0.0	7.2–15.1	G
Basophils (10^3^/μL)	23	0.0	0.0	0.0	0.0	0.0	0.0	0.0	G
RBCs (10^6^/μL)	23	3.0	0.8	1.4	4.6	1.3–4.6	0.8–1.8	4.1–5.1	G
PCV (%)	25	38.7	4.7	30.0	49.0	28.9–48.5	26.3–31.9	45.7–51.3	G
Hb concentration (g/dL)	24	14.5	2.5	11.4	19.3	9.3–19.7	7.8–10.8	18.1–21.2	G
Mean corpuscular volume (fL)	23	140.5	38.4	96.8	259.3	59.3–221.8	40.8–84.6	181.0–256.2	G
Mean corpuscular hemoglobin (pg)	23	51.8	16.1	34.2	104.0	17.7–86.0	9.7–28.6	69.3–107.7	G
Mean corpuscular hemoglobin concentration (g/㎗)	24	37.1	5.1	27.1	47.1	26.4–47.9	23.5–29.5	44.5–50.9	G

N, number (sample size); WBC, white blood cell; RBC, red blood cell; PCV, packed cell volume; Hb, hemoglobin; MCV, mean corpuscular volume; MCH, mean corpuscular hemoglobin; MCHC, mean corpuscular hemoglobin concentration; SD, standard deviation; Min, minimum; Max, maximum; RI, reference interval; L, 90% confidence interval for lower limit; U, 90% confidence interval for upper limit; D, distribution; G, Gaussian; NG, non-Gaussian.

**Table 14:** Clinical chemistry reference intervals in Eurasian magpies.

Parameter (SI units)	N	Mean	SD	Min	Max	RI	D
Total protein (g/㎗)	11	3.2	0.5	2.4	4.1	2.4–3.8	G
Potassium (mmol/L)	11	4.5	0.9	2.7	5.5	2.7–5.3	G
Calcium (㎎/㎗)	11	9.6	0.8	8.5	10.7	8.5–10.4	G
Phosphorus (㎎/㎗)	11	3.9	1.4	1.8	6.4	1.8–5.3	G
Sodium (mmol/L)	11	157.0	3.4	149.0	161.0	149.0–160.0	G
Albumin (g/㎗)	11	2.2	0.4	1.5	2.7	1.5–2.5	G
Globulin (g/㎗)	11	1.0	0.4	0.3	1.4	0.3–1.3	NG
AST (IU/L)	11	794.6	524.0	0.0	1807.0	0.0–1172.0	G
Glucose (mg/dL)	11	371.9	132.4	243.0	651.0	243.0–597.0	G
CK (IU/L)	11	1603.7	2253.8	262.0	8166.0	262.0–2029.0	NG
Uric acid (mg/dL)	10	7.2	4.8	2.1	15.2	2.1–14.8	G
Bile acid (μmol/L)	11	30.0	10.4	0.0	34.0	<35	NG

N, number (sample size); AST, aspartate aminotransferase; CK, creatine kinase; SD, standard deviation; RI, reference interval; D, distribution; G, Gaussian; NG, non-Gaussian.

## 4 Discussion

Researching hematological and clinical chemistry reference intervals in wild birds poses numerous challenges and difficulties. Acquiring blood samples directly from animals living in the wild, especially those not captured, can be particularly challenging. Therefore, research focusing on patients that have been injured, rescued, and then released back into the wild has significance because it reflects the real-life situations in the field and can enable the derivation of realistic reference intervals. For those reasons, obtaining hematologic and clinical chemistry reference intervals for six species commonly rescued in the Republic of Korea is significant.

Previous studies published hematologic and clinical chemistry reference intervals for Eurasian eagle owls (13, 14), but they differed from this study in that they analyzed non-releasable birds or focused on nesting birds. This study analyzed pre-release samples from clinically healthy Eurasian eagle owls to estimate hematologic reference intervals that can be clinically used for a broad range of parameters through comparison with existing reference data. The Eurasian eagle owl was characterized by significantly lower RBC counts and higher MCV, MCH, and MCHC than all the other species except the brown hawk-owl. Its levels of calcium and phosphorus were higher than those in other species.

The Oriental turtle dove, domestic pigeon, and Eurasian magpie are the most commonly rescued birds in the Republic of Korea (15). The hematologic and clinical chemistry reference intervals needed to diagnose and treat these rescued wild birds have been lacking. Although hematologic and clinical chemistry reference intervals for the Oriental turtle dove and domestic pigeon have been established in other countries (16, 17), no previous study of those species has examined individuals from the Republic of Korea. Total protein in the Oriental turtle dove and domestic pigeon were lower than in the Eurasian eagle owl and brown hawk-owl. Considering the limited clinical chemistry information available for the Oriental turtle dove in this study, this difference was found to be significant even when compared with existing data (16). Carnivores typically have higher total protein levels than herbivores, and that principle might also apply to birds (18). It is remarkable that the eosinophil levels in Eurasian magpies were higher than in any of the other species in this study. Although it might be challenging to directly apply the clinical chemistry reference intervals developed here because no previous research to rely on exists, the results of this study can be a reference point for further development.

Treating migratory birds, such as the brown hawk-owl, at wildlife rescue centers poses more time constraints than those faced when treating non-migratory birds. Hematologic studies have been conducted to aid in the rapid diagnosis of other Strigidae species (19, 20). However, research on hematologic and clinical chemistry reference intervals for the brown hawk-owl had not yet been conducted. Compared with the Eurasian eagle-owl, another Strigidae species, the hematologic reference intervals did not differ significantly. In the clinical chemistry reference intervals, phosphorus levels were significantly lower in the brown hawk-owl. Although it is true that hematologic and clinical reference intervals can vary even within the same family, determining whether such variations are statistically significant is a separate issue.

Although the hematologic and clinical chemistry reference intervals for the common kestrel had been studied in another country, it is difficult to apply those results directly to medical practice due to limitations in the hematological parameters (21). The previous study had a sample size of 16, whereas this study used a larger sample (*N* = 76), to offer higher reliability and consistency, especially when the new reference interval includes the previous one. This study can thus be considered to strengthen and expand the existing results by targeting a larger sample size and a wider range of hematological and clinical chemistry parameters than those previously published. Even when considering sample size and existing research, the hematologic and clinical chemistry reference intervals for the common kestrel presented here will provide a more accurate and effective guideline for diagnosis and treatment. The lower WBC and heterophil counts in the common kestrel, compared with other species, is highly characteristic and reliable.

In this study, the reference intervals could be somewhat wider. Reference intervals can be affected by sex and age (22), and this study did not investigate sex or age. It is difficult to know the exact age and sex of free-living wild birds rescued at a wildlife rescue center without additional testing (23, 24). These reference intervals made for clinically healthy adult wild birds provide useful information for clinicians working in wildlife rescue centers.

This study has newly established or supplemented the hematological and clinical chemistry profiles of six wild birds species commonly rescued in the Republic of Korea. Because it targets wild birds rescued and treated at wildlife rescue centers, it is expected to serve as a useful criterion for diagnosing and treating patients at such centers.

## Data Availability

The original contributions presented in the study are included in the article/supplementary material, further inquiries can be directed to the corresponding author.
